# Supplementary material for: Warm rings in mesoscale eddies in a cold straining ocean
Source: Nat Commun. 2025 Oct 20;16:9252. doi: 10.1038/s41467-025-64308-y (PMC12537925; doi:10.1038/s41467-025-64308-y)
Supplement: Supplementary file 1 — Supplementary Information [file 41467_2025_64308_MOESM1_ESM.pdf]

Supplementary Information for

Warm Rings in Mesoscale Eddies in a Cold Straining Ocean

Authors:

Huizi Dong[1,2,3,*], Meng Zhou[1,2,3,*], James C. McWilliams[4], Roshin P. Raj[5], Francesco d'Ovidio[6], Ilker Fer[7], Lixin Qu[1,2,3], Bo Qiu[8], Lia Siegelman[9], Zhengguang Zhang[10,11], Walker O. Smith, Jr.[1,2,3], Ann Kristin Sperrevik[12]

Affiliations:

[1] Key Laboratory of Polar Ecosystem and Climate Change, Ministry of Education and School of Oceanography, Shanghai Jiao Tong University, Shanghai, China

[2] Shanghai Key Laboratory of Polar Life and Environment Sciences, Shanghai Jiao Tong University, Shanghai, China

[3] Shanghai Frontiers Science Center of Polar Science, Shanghai Jiao Tong University, Shanghai, China

[4] Department of Atmospheric and Oceanic Sciences, University of California, Los Angeles, CA, USA

[5] Nansen Environmental and Remote Sensing Center, Norway and Bjerknes Center for Climate Research, Bergen, Norway

[6] Sorbonne Université, CNRS, IRD, MNHN, Oceanography and Climate Laboratory: Experiments and Numerical Approaches (LOCEAN-IPSL), Paris, France

[7] Geophysical Institute, University of Bergen and Bjerknes Center for Climate Research, Bergen, Norway

[8] Department of Oceanography, University of Hawaii at Manoa, Honolulu, Hawaii, USA

[9] Scripps Institution of Oceanography, University of California, San Diego, La Jolla, CA, USA

[10] Key Laboratory of Physical Oceanography, Frontier Science Center for Deep Ocean Multispheres and Earth System (FDOMES), Ocean University of China, Qingdao, China

[11] Laoshan Laboratory, Qingdao, China

[12] Division for Ocean and Ice, Norwegian Meteorological Institute, Oslo, Norway

[*] Correspondence to:

Huizi Dong (huizidong@sjtu.edu.cn), Meng Zhou (meng.zhou@sjtu.edu.cn)

Inventory of Supplementary Information:

Supplementary Notes

Supplementary Table S1

Supplementary Figures S1–S14

Supplementary References

Supplementary Notes

Quasi-geostrophic frontogenesis theory and vertical velocity. From the perspective of frontogenesis, the quasi-geostrophic theory can be written in terms of the equations of conservative momentum, buoyancy, and continuity, respectively, as follow[1,2]:

$$D_g u_g = f v_a,\ D_g v_g = -f u_a \tag{1}$$

$$\phi_z = b \tag{2}$$

$$D_g b = -N^2 \omega \tag{3}$$

$$u_{a,i}^{i} + \omega_z = 0, \tag{4}$$

where the subscripts g and a represent geostrophic and ageostrophic components, respectively, $u_i = (u, v)$, and $D_g[\cdot] = [\cdot]_t + u_g^j[\cdot]_j$ is the geostrophic advective time derivation. The first two equations are obtained by subtracting the geostrophic balance term from the horizontal-momentum equations. The buoyancy gradient equation is obtained by taking the horizontal derivative of the Equation (3) as follows:

$$D_g b_i = Q^i - N^2 \omega_i, \tag{5}$$

where $Q^i \equiv -u_{g,i}^j b_j$ is known as the Q-vector[3,4], serving as the primary forcing for deformation frontogenesis. The omega equation for vertical velocity w can be derived by eliminating time derivatives from Equations (1)-(4) and incorporating the Q-vector divergence with the thermal wind balance and continuity constraint[3-5]:

$$N^2 w_{ii} + f^2 w_{zz} = 2Q_i^i \tag{6}$$

Frontogenesis develops in a 2D surface front $b(x, z)$ under the combined influence of a barotropic deformation flow (strain rate u_x) and a surface-intensified geostrophic along-front current. In this 2D configuration, the Q-vector divergence on the right-hand side of the omega

equation (Equation 6) reduces to $-2(u_x b_x)_x$, which is negative on the less dense side of the front ($b_{xx} > 0$) and positive on the dense side ($b_{xx} < 0$). Given the elliptic nature of the omega operator on the left-hand side, the solution for w tends to have signs opposite to the forcing term, yielding upward motion (w>0) on the less dense side and downward motion (w<0) on the dense side. The vertical velocity can be scaled from these relations as:

$$w \sim Ro_d \frac{Vh}{L} \tag{7}$$

where $Ro_d = u_x/f$ is the deformation Rossby number, V is the characteristic geostrophic velocity, h is the mixed layer depth, and L is the frontal width. Following these relations, frontogenesis intensification enhances vertical velocity (w) through increased strain rate (u_x) and narrowed frontal width (L). For typical frontal conditions (Ro_d~ 0.1-0.2, V ~ 0.1-1 m s^{-1}, h ~ 10-100 m, L ~ 1-10 km), this scaling yields $w \sim O(10^{-3}$ m $s^{-1})$, which is consistent with our glider-observed vertical velocities (~60 m d^{-1}) at eddy-fronts (Fig. 2**g**). This theoretical estimate confirms that the observed vertical velocities along the eddy edges can be well explained by the frontogenesis processes driven by submesoscale dynamics.

Quality control and calibration of Seaglider data. The dataset includes temperature and salinity profiles linearly interpolated at 1-dbar intervals, with two profiles collected per dive (one during descent and one during ascent). In situ compass calibrations were conducted at sea during the early stages of each deployment. Data processing was performed using the University of East Anglia's Seaglider Toolbox[6,7], based on standard Seaglider basestation routines. Thermal lag corrections for the conductivity sensor followed the methodology described by Garau et al.[6], and flight model regressions were conducted following Frajka-Williams et al.[7].

Temperature and salinity calibrations for the NACO missions (M1–6) were conducted using available hydrographic data provided by the Institute of Marine Research in Bergen, Norway, comprising 253 casts collected from July 2012 to 2016 within the region bounded by 69–71°N and 0–15°E. Calibration offsets (Supplementary Tab. S1) were determined by applying a least-squares fit to glider data collected between depths of 700 m and 1000 m, using a reference linear temperature-salinity (T/S) relationship representing the mixing line between Atlantic Water and deeper waters below 700 m. Temperature and salinity calibrations for the ProVoLo missions (M7–8) were performed by comparing the glider measurements to hydrographic surveys conducted within the framework of the ProVoLo project. Temperature and salinity offset values (Supplementary Tab. S1) were obtained by comparing glider data collected in the weakly stratified core of the Lofoten Basin Eddy with calibrated shipborne CTD profiles measured within the same eddy during three PROVOLO cruises conducted in June 2016, March 2017, and September 2017. For all gliders, temperature data are assumed accurate to within 0.005 °C, based on the manufacturer's specified absolute calibration accuracy (0.002 °C) and drift (0.0002 °C $month^{-1}$).

Quality control procedures included applying strict thresholds to salinity and temperature (separately for the upper 200 m and deeper layers), as well as identifying and excluding density inversions below the mixed layer exceeding -0.001 kg m^{-3} over 15 m. Data gaps smaller than 15 m were linearly interpolated.

Analysis of the Seaglider data. We used observational data from eight Seaglider missions (M1–M8). Seagliders are autonomous underwater vehicles that profile the ocean by adjusting buoyancy, enabling multi-month missions[6-9]. Raw measurements were collected at 10–32 s

intervals with vertical velocities of 7–10 cm s^{-1}, yielding a native vertical resolution of 1–3 m. A key challenge in using glider data for submesoscale studies is potential contamination from high-frequency oceanic phenomena like internal waves, which manifests as two distinct effects: Doppler smearing and aliasing. Doppler smearing is a consequence of the glider's finite speed through the water (~0.2 m s^{-1}) and manifests as a smearing of the true wave number and frequency[10]. In contrast, aliasing is a function of the sampling rate, which causes variability from higher wave numbers to be folded into the resolved lower wave numbers[10].

To mitigate these effects, we used objectively mapped fields in our analyses. The along-track distance was first calculated using the trajectory estimates from the glider's flight model and smoothed using a Gaussian window with a 10 km scale. The average position, time, and along-track distance were then determined for each dive and climb trajectory. Depth-average-current (DAC) estimates[11,12] between the consecutive surfacings were interpolated to these averaged times and assumed to remain constant throughout each individual profile. Before calculating buoyancy, profiles were vertically binned into intervals of 5 m, and potential density was adjusted for each profile to ensure gravitational stability. Buoyancy gradients[13] were computed after smoothing the buoyancy field using a two-dimensional Gaussian window with vertical and horizontal scales of 25 m and 10 km, respectively. Thermal wind shear was then derived from the dynamic height calculated from these smoothed fields to filter out small-scale oscillations caused by unbalanced flows and internal waves.

Next, the data were averaged into vertical bins of 5 m and along-track bins of 2 km in preparation for objective interpolation. Empty bins were left as NaNs to avoid introducing

bias from uneven spatial sampling, especially in areas of weak currents. Subsequently, the binned data were objectively mapped using a Gaussian correlation function, with correlation length scales of 10 km (horizontal) and 25 m (vertical), and an error variance of 0.05, onto a regular grid with 2 km horizontal and 5 m vertical resolution. Density was recalculated from the objectively mapped temperature and salinity, and profiles were vertically sorted to eliminate potential density inversions introduced by mapping. Finally, mapped values with an error-to-signal variance ratio exceeding 0.5 were masked.

Despite the 10 km –25 m smoothing, strong lateral buoyancy gradients and vertical velocities remain evident at eddy edges (Supplementary Fig. S14), demonstrating the intensity of these submesoscale signals. Furthermore, these features exhibit physically consistent spatial structures and patterns. Our analysis of all glider missions (M1–M8) indicates that vertical velocities and vertical heat transport (VHT) are underestimated by approximately 15–20% in the smoothed fields, compared with the 2-km bin averaged, unsmoothed results.

Validation using along-isopycnal analysis. Rudnick et al.[10] demonstrated that analysis of variables measured along isopycnals effectively reduced noise from internal waves and Doppler smearing and aliasing effects. To further validate our results, we conducted an isopycnal analysis by projecting temperature and salinity data from depth coordinates onto isopycnal surfaces. The resulting map of potential temperature on isopycnals revealed the thermal structure of two anticyclonic eddies and one cyclonic eddy transited by the seaglider, with strong temperature gradients at the eddy edges (Supplementary Fig. S11**d**). To facilitate the diagnosis of vertical velocity via the omega equation, we smoothed the isopycnal depths

and on-isopycnal T/S fields with a 10 km low-pass Gaussian filter to mitigate wave-induced undulations before reconstructing them into depth coordinates.

The resulting fields of lateral buoyancy gradient (b_x) and vertical velocity (w) were found to be in strong agreement, both in spatial structure and location, with those obtained from our spatially filtered z-coordinate analysis (Supplementary Fig. S11**b**,**c**,**e**,**f**). This agreement indicates that our processing effectively suppresses contamination from internal waves and aliasing, enabling the reliable extraction of strong submesoscale signals at mesoscale eddy edges. Furthermore, the spatial patterns and magnitudes of these features are consistent with our model validation, established submesoscale dynamics, and values reported in the literature[14,15].

Comparison between mesoscale and submesoscale VHT. To compare the effects of submesoscale and mesoscale processes on VHT, we extracted both submesoscale and mesoscale components of temperature and vertical velocity from the cross-basin M1 transect measured by the Seaglider (Supplementary Fig. S8). The submesoscale components of temperature (T'_{sub}) and vertical velocity (w'_{sub}) were obtained using a bandpass filter for scales of 2–15 km, while the mesoscale components (T'_{meso} and w'_{meso}) were extracted using a bandpass filter for scales of 20–100 km[16,17]. Thus, the submesoscale and mesoscale VHT can be calculated as follows:

$$VHT_{sub} = \rho C_p w'_{sub} T'_{sub} \quad (8)$$

$$VHT_{meso} = \rho C_p w'_{meso} T'_{meso} \quad (9)$$

The results reveal that the submesoscale vertical velocity (~60 m d^{-1}), predominantly located at the eddy edges, significantly exceeds the mesoscale vertical velocity (<10 m d^{-1}), which is

distributed across both the eddy edges and interiors (Supplementary Fig. S8**d**,**e**). These submesoscale motions generate VHT$_{sub}$ of up to 1400 W m^{-2}, with upward heat transport dominating. In contrast, VHT$_{meso}$ is not only an order of magnitude lower than VHT$_{sub}$, but also exhibits both positive and negative values, indicating that the net heat flux from VHT$_{meso}$ is even lower (Supplementary Fig. S8**f**,**g**).

We further extended our analysis by decomposing temperature and vertical velocity transects from all Seaglider measurements (transects M1-8, 2012-2017; Fig.1) into submesoscale and mesoscale components, subsequently calculating corresponding heat transports (VHT$_{sub}$ and VHT$_{meso}$) for each transect. All four parameters (w'_{sub}, w'_{meso}, VHT$_{sub}$, and VHT$_{meso}$) from each transect were collocated onto a normalized eddy-centric coordinate system based on their relative positions to eddies, and then composited separately (Supplementary Fig. S7). Comparison between the composite results of submesoscale and mesoscale components (Figs. 3**e**,**i**, and Supplementary Fig. S9) indicates that strong w'_{sub} are predominantly confined at eddy edges, whereas w'_{meso} are distributed across both eddy edges and interiors, with magnitudes nearly an order of magnitude lower. Regarding heat transport, VHT$_{meso}$ is approximately 1/6 to 1/5 of VHT$_{sub}$ without considering net heat fluxes; however, when net heat fluxes are included, VHT$_{sub}$ exceeds VHT$_{meso}$ by an order of magnitude.

Numerical simulation verification. To further validate the vertical velocity fields and vertical heat transport (VHT) derived from the observational data, we compared these results with high-resolution numerical simulations performed using the Regional Ocean Modeling System (ROMS) in the Lofoten Basin area[18,19]. The model has a horizontal resolution of 2.4 km and 42 vertical layers, and it is forced by 3-hourly operational forecast data from the

Integrated Forecast System of the European Centre for Medium-Range Weather Forecasts (ECMWF). Air-sea fluxes for momentum and heat are calculated using the CORA 3.0 bulk flux algorithms with ROMS[20]. Open boundary conditions consist of daily averages of all state variables from the TOPAZ4 reanalysis[21]. Since the TOPAZ4 does not include the inverse barometer effect, the sea surface elevation and currents are adjusted to account for the local atmospheric pressure. Harmonic tidal forcing, consisting of eight tidal constituents from the TPXO9 global inverse barotropic model, is also imposed along the open boundaries[22]. Additionally, the model assimilates satellite sea surface temperature (SST), in-situ observations from Argo, drifters, CTD sections, ferry boxes, and HF-radar surface currents and is described in previous studies[18,19].

The sea level anomaly (SLA) and finite-size Lyapunov exponent (FSLE) fields in the Lofoten Basin, along with daily averaged vertical velocity and VHT profiles from numerical simulations, are presented in Supplementary Figs. S5,6. The simulation results along three transects (two meridional transects T1 and T3, and one zonal transect T2) reveal intense vertical velocities exceeding 40 m d^{-1} at the eddy edges, spanning horizontal widths of approximately 3–8 km. These vertical currents extend well beyond the mixed layer, consistent with observational results presented in the main text. Along the meridional transect T1 at approximately 7.5–8°E (Supplementary Fig. S5) and at the boundary of eddy ACE-04 along transect T3 (Supplementary Fig. S6), cross-frontal secondary circulation notably flattens the isopycnal surfaces. Additionally, transect T2 crosses two anticyclonic eddies zonally, and Supplementary Fig. S5**b**–**e** presents the temperature and vertical velocity fields at depths of 50 m and 250 m. Results show that vertical velocities exceeding 30 m d^{-1} occur where

temperature gradients are strongest along eddy edges. These vertical velocities exhibit a dinstinct paired structure of upward and downward flows, indicative of a strong secondary circulation at the eddy edges. The simulated VHT predominantly shows positive (upward) heat transport at the eddy edges, with maximum values reaching up to 2000 W m^{-2}.

To further verify whether these intense vertical motions at eddy edges are primarily governed by submesoscale processes, we conducted additional analyses using outputs from the Norkyst-DA model. These analyses span the period from December 1, 2017, to November 30, 2019, and examine the relative contributions of mesoscale and submesoscale processes to VHT (Supplementary Fig. S10). Submesoscale components (w'_{sub} and T'_{sub}) were derived by applying a spatial high-pass filter (0.2° latitude × 0.5° longitude half-power filter cutoffs) to the daily vertical velocity and temperature fields from the model. Mesoscale components (w'_{meso} and T'_{meso}) were obtained by subtracting the annual mean from each grid point, followed by low-pass filtering using the same spatial cutoffs. Prior to compositing analyses (see Methods), we calculated VHT_{sub} and VHT_{meso} using Equations (8) and (9), respectively. The modeling results further confirm that the strong vertical motions at eddy edges are predominantly driven by submesoscale processes. Within the eddy-centered normalized coordinate system, VHT_{meso} amounts to approximately one-eighth of the VHT_{sub}, even without considering the net heat transport.

The above case studies, combined with the two-year composite statistical analysis, demonstrate consistent agreement between numerical simulations and observations regarding the structure, depth range, and intensity of vertical velocities and VHT (Supplementary Figs. S5,6,10). These results reinforce the findings of our study and further highlight the significant

influence of mesoscale eddy-associated submesoscale processes on oceanic vertical heat transport in regions characterized by intense air-sea interaction, such as the Nordic Seas.

Supplementary Tables

Supplementary Tab. S1| Details of the Seaglider deployments (M1-M8)

Mission	Seaglider	Deployed	Recovered	Duration (days)	Dives	Salinity correction (g kg^{-1})	Temperature correction (K)
M1	sg559	4 Jul 2012	25 Jan 2013	205	617	+0.0072	-
M2	sg562	14 Feb 2013	16 Sep 2013	214	680	+0.0046	-
M3	sg561	11 Oct 2013	17 Feb 2014	129	457	-0.0023	-
M4	sg563	26 Feb 2014	1 Nov 2014	248	831	+0.0183	-
M5	sg562	18 Dec 2014	18 Jul 2015	91	91	+0.0022	-
M6	sg559	18 Dec 2014	18 Jul 2015	212	699	-0.0100	-
M7*	sg561	4 May 2016	13 Jan 2017	253	818	+0.0040	+0.006
M8**	sg563	13 Jan 2017	24 Jul 2017	191	649	-	+0.012

* Excluded 2 profiles with >20% density inversions.

**Due to a technical issue temperature and salinity were not recorded for the last 17 days of the mission.

- indicates no correction was applied.

Supplementary Figures

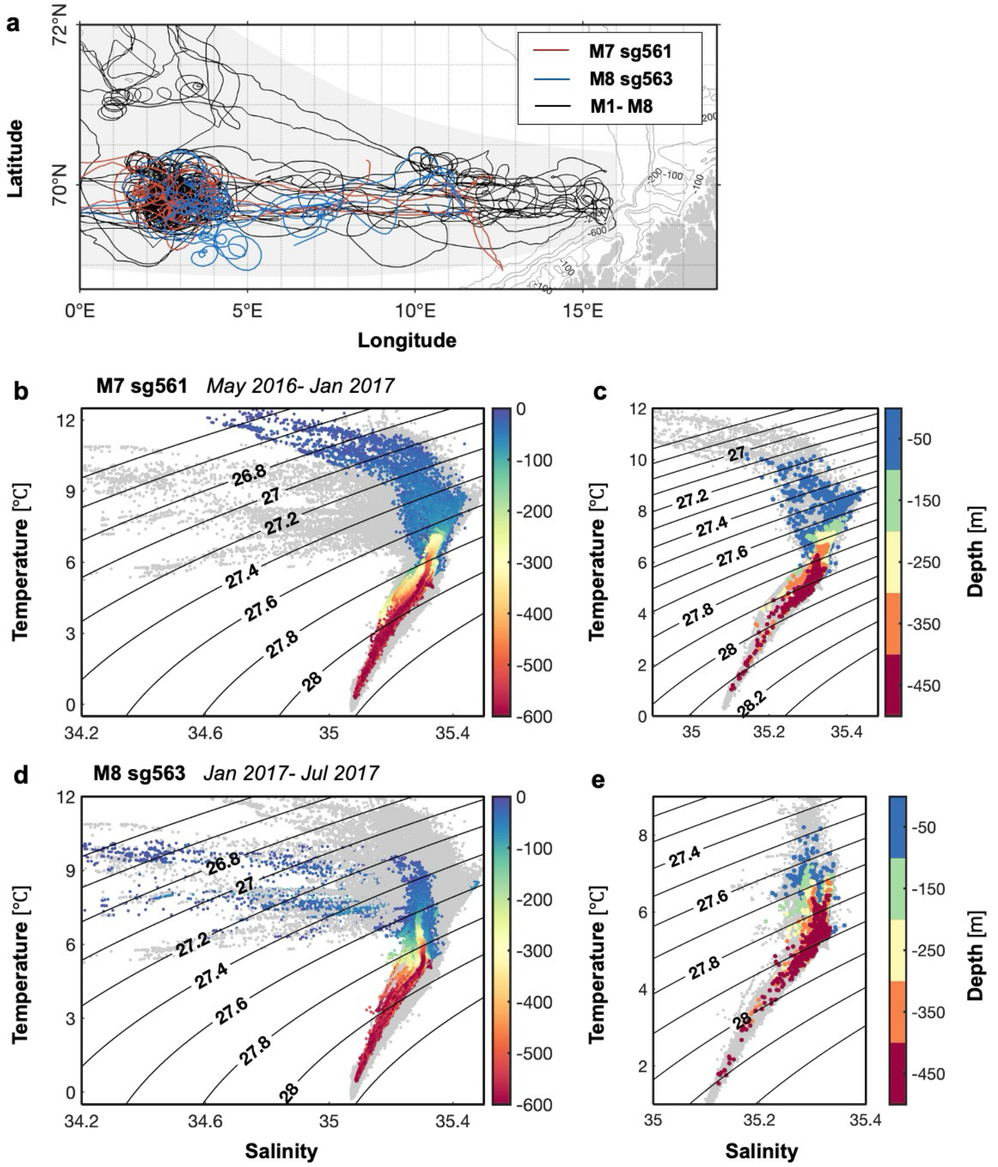

Supplementary Fig. S1 | Trajectories of eight Seaglider missions (M1-M8; black lines), with missions M7 and M8 highlighted in red and blue, respectively. **b**, **d**, T-S distributions across depths from 0 to 600 m for missions M7 (**b**) and M8 (**d**), plotted against the background (grey points) of all Seaglider missions (M1-M8). **c**, **e**, Similar to **b**, **d** but showing T-S distributions only at five depth levels (50, 150, 250, 350, and 450 m).

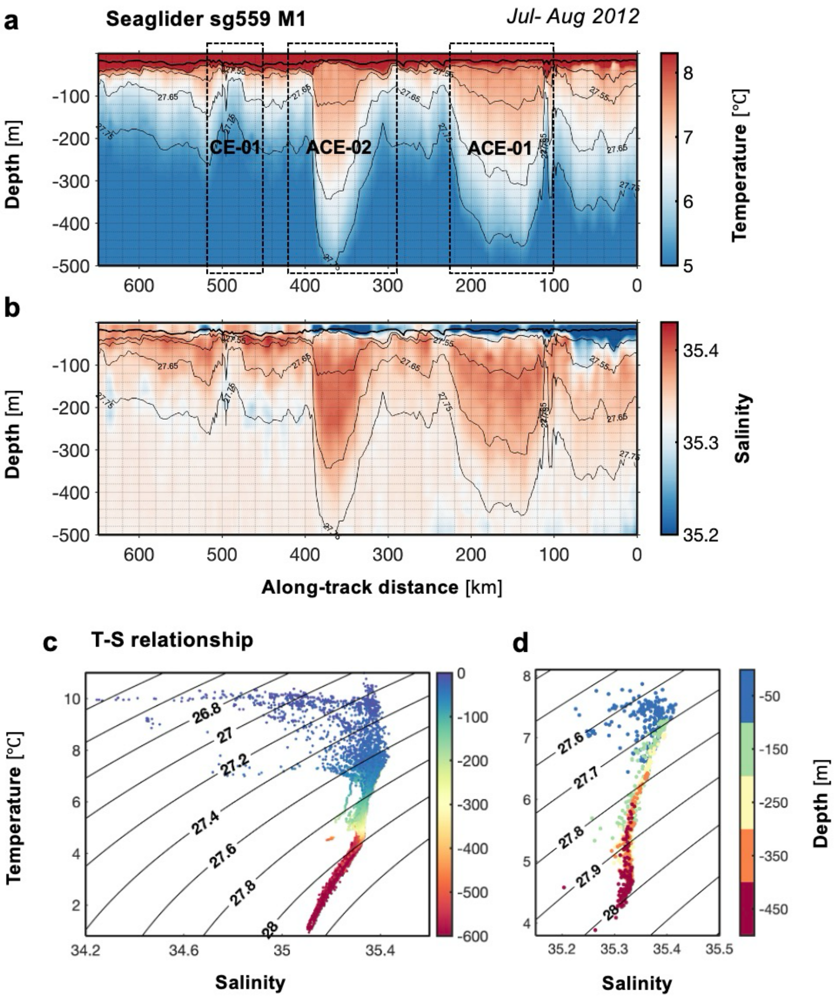

Supplementary Fig. S2 | Hydrographic characteristics and T–S distribution along the Seaglider transect from 6 July to 16 August 2012. Vertical sections showing **a** temperature and **b** salinity obtained from Seaglider measurements (same transect as in Fig. 2 of the main text). **c** T-S distribution across depths of 0-600 m along the transect. **d** T-S distribution similar to **c** but show only for five depth levels (50, 150, 250, 350, and 450 m).

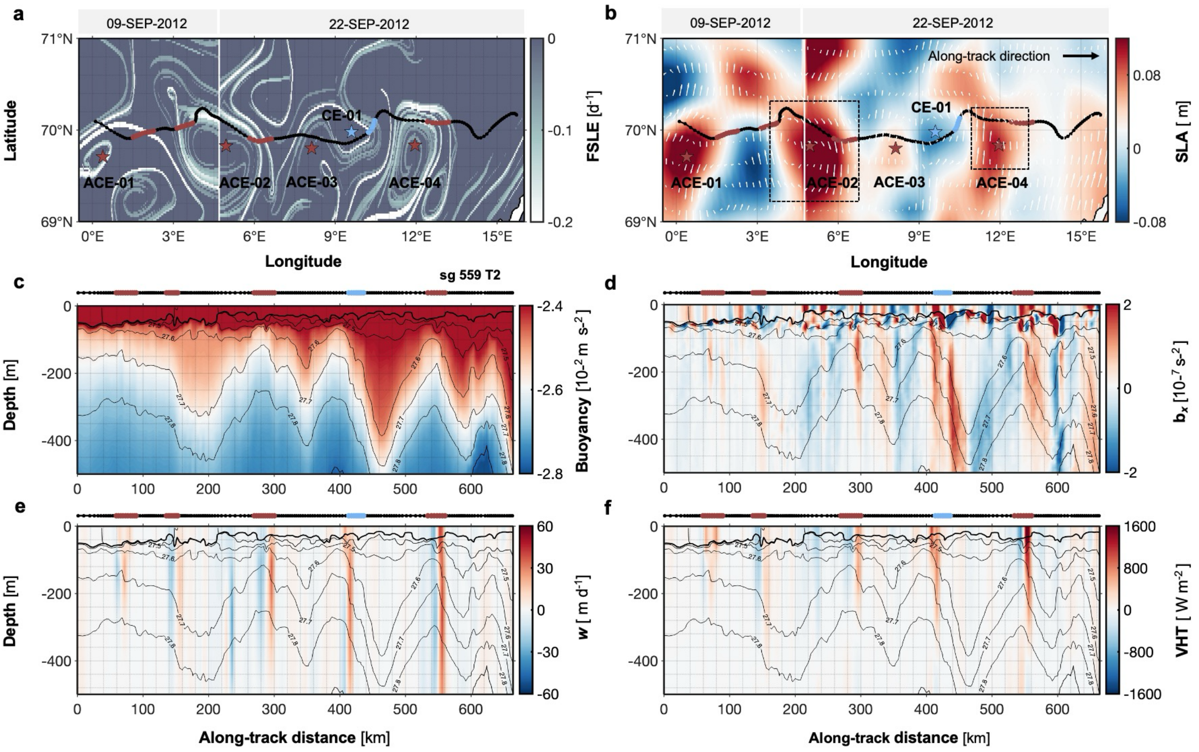

Supplementary Fig. S3 | a Finite-size Lyapunov exponent (FSLE), **b** sea level anomaly (SLA) superimposed on the Seaglider track from 6 September 2012 to 10 October 2012. The glider's vertical section of **c** buoyancy, **d** lateral buoyancy gradient (b_x), **e** vertical velocity (w). **f** The glider's vertical section of vertical heat transport (VHT), with positive (negative) values denoting upward (downward) heat transport. The mixed layer depth (MLD) is shown by a thick black line in **c**-**f**. In panels **a** and **b**, red and blue dots indicate where the Seaglider traversed the edges of four anticyclonic eddies (ACEs) and one cyclonic eddy (CE), respectively, corresponding to the same color-coded positions along the Seaglider track displayed above panels **c**-**f**.

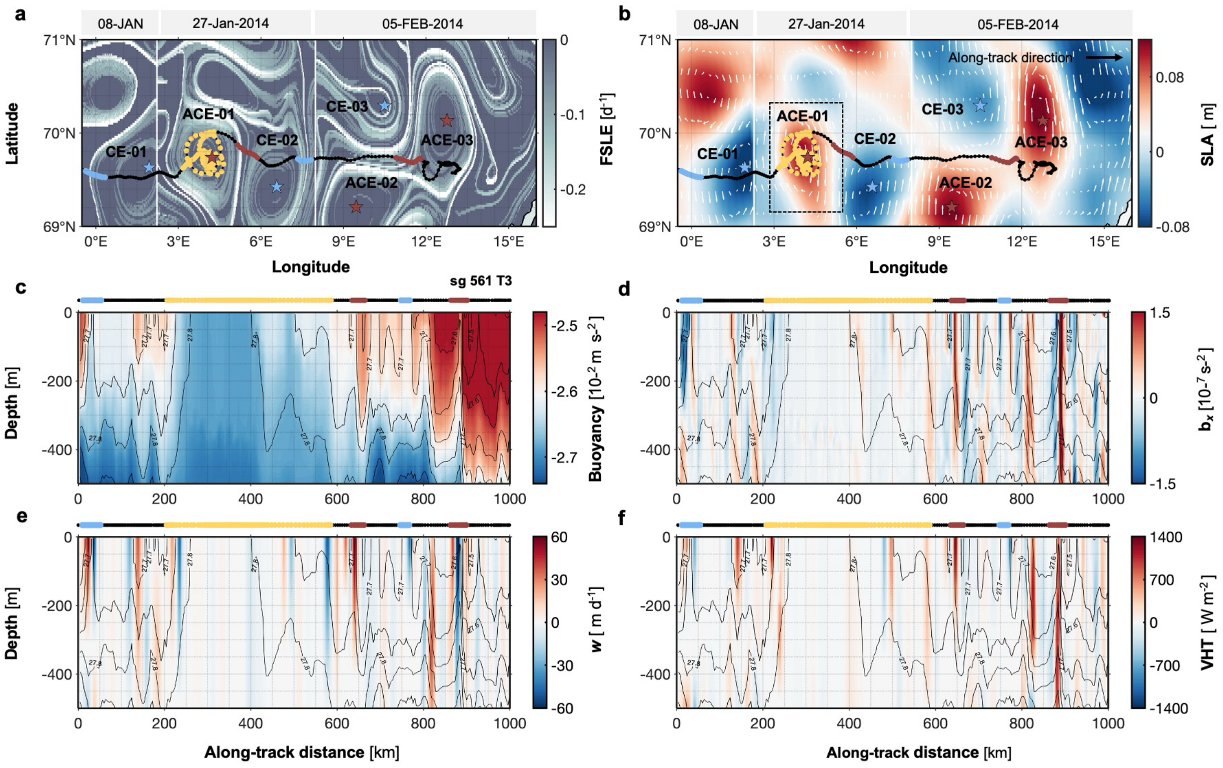

Supplementary Fig. S4 | a Finite-size Lyapunov exponent (FSLE), **b** sea level anomaly (SLA) superimposed on the Seaglider track from 6 January 2014 to 17 February 2014. The glider's vertical section of **c** buoyancy, **d** lateral buoyancy gradient (b_x), **e** vertical velocity (w). **f** The glider's vertical section of vertical heat transport (VHT), with positive (negative) values denoting upward (downward) heat transport. In panels a and b, red and blue dots indicate where the Seaglider traversed the edges of three ACEs and two CEs, respectively, with the glider remaining within one anticyclonic eddy (ACE-01) for 14 days (marked by yellow dots). These dots correspond to the same color-coded positions along the Seaglider track displayed above panels **c-f**.

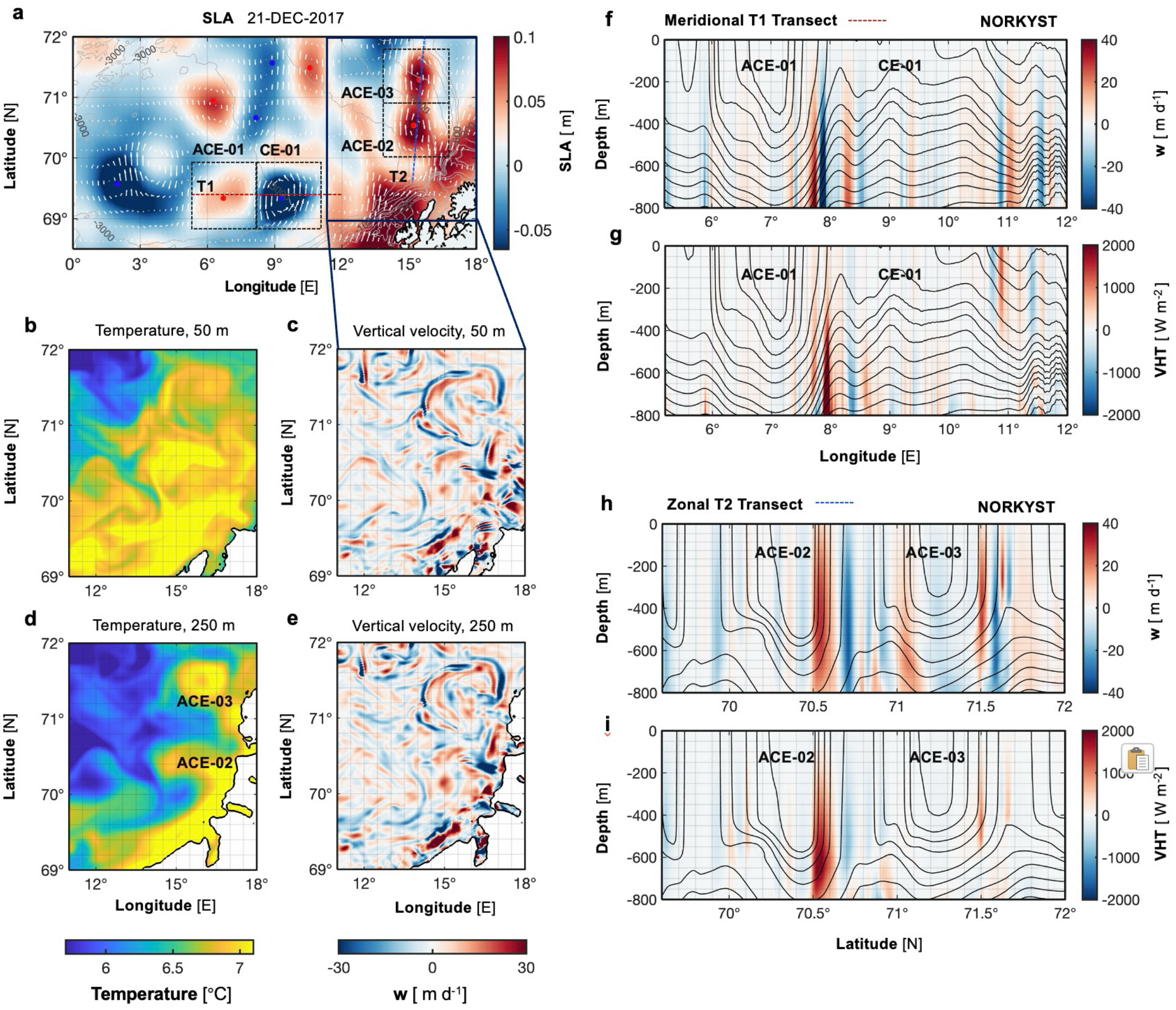

Supplementary Fig. S5 | Vertical velocities and vertical heat transport (VHT) derived from high-resolution numerical simulation. **a** Sea Level Anomaly (SLA) and **b** Finite-Size Lyapunov Exponent (FSLE) fields on 21 December 2017, with identified ACEs (red dots) and CEs (blue dots). Panels **b-e** show properties within the region indicated by the black solid box in panel **a**: temperature at **b** 50 m and **d** 250 m depth, and vertical velocity at **c** 50 m and **e** 250 m depth. Vertical transects from the numerical simulation along T1 (red dashed lines in panels **a** and **b**; 69.4°N, 5.2°E–12°E) showing **g** vertical velocities and **h** VHT, and along T2 (blue dashed lines in panels **a** and **b**; 69.6°N, 15.3°E–72°N, 15.8°E) showing **i** vertical velocities and **j** VHT. Black contours in panels **g–j** represent isopycnals.

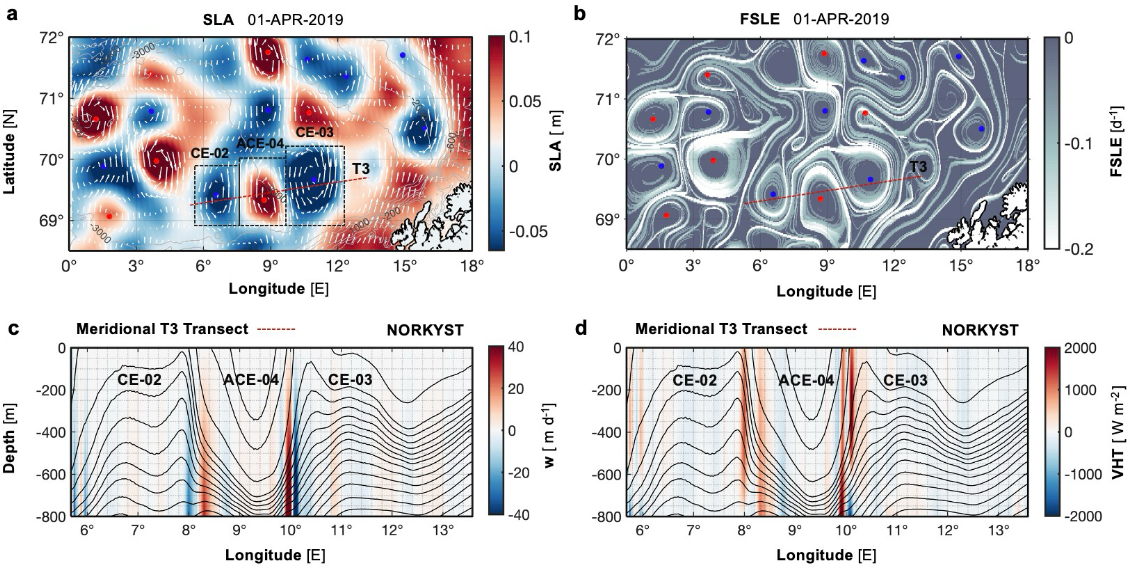

Supplementary Fig. S6 | Vertical velocities and vertical heat transport (VHT) from the high-resolution numerical simulation. **a** Sea Level Anomaly (SLA) and **b** Finite-Size Lyapunov Exponent (FSLE) fields superimposed on the identified ACEs (red dots) and CEs (blue dots) for 01 April 2019. Vertical transect of T3 (indicated by blue dashed lines in panels **a** and **b**) from the high-resolution numerical simulation at 69.3°N, 5.7°E to 69.7°N, 13.6°E, showing **c** Vertical velocities and **d** VHT. Isopycnals are shown by black lines in panels **c-d**.

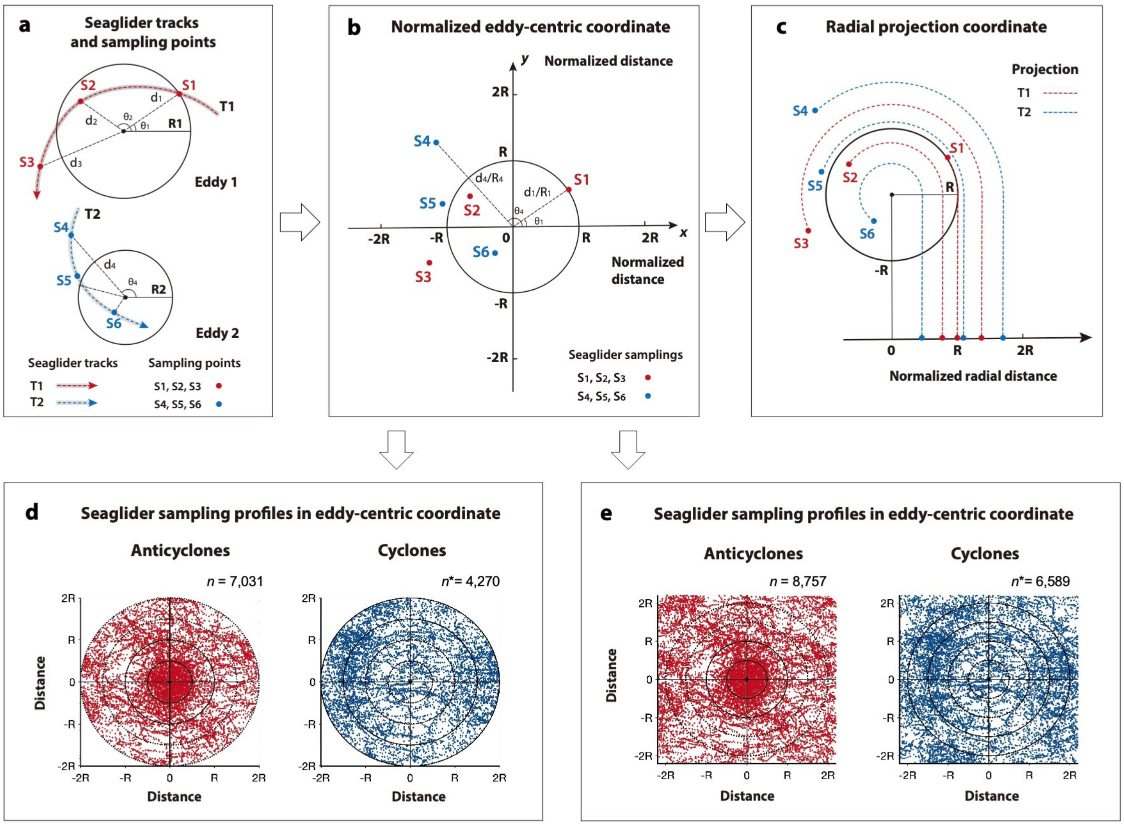

Supplementary Fig. S7 | Schematic summarizing the process of collocating Seaglider sampling profiles onto a normalized, eddy-centric coordinate system. a for each Seaglider trajectory through an eddy, sampling profiles within two radii from the eddy center were identified. The location of each profile (S1-S6) relative to the eddy center is defined as D (d, θ), where d represents the radial distance normalized by the eddy radius (d/R_x) and θ is the azimuthal angle relative to due east. **b** All profiles (S1-S6) were then collocated onto a common normalized eddy-centric coordinate system, expressed in units of dimensionless eddy radius (R). **c** Profiles in the normalized eddy-centric coordinates (**b**) were projected onto a radial distance range of [0, 2R]. **d** Spatial distribution of all Seaglider sampling profiles collected within anticyclonic and cyclonic eddies in the Lofoten Basin (Fig. 1**b**) between 4 July 2012 and 9 August 2017. n and $n*$ indicate the number of Seaglider sampling profiles associated with anticyclonic and cyclonic eddies, respectively. **e** Same as **d**, except for the

range of normalized eddy-centric coordinate.

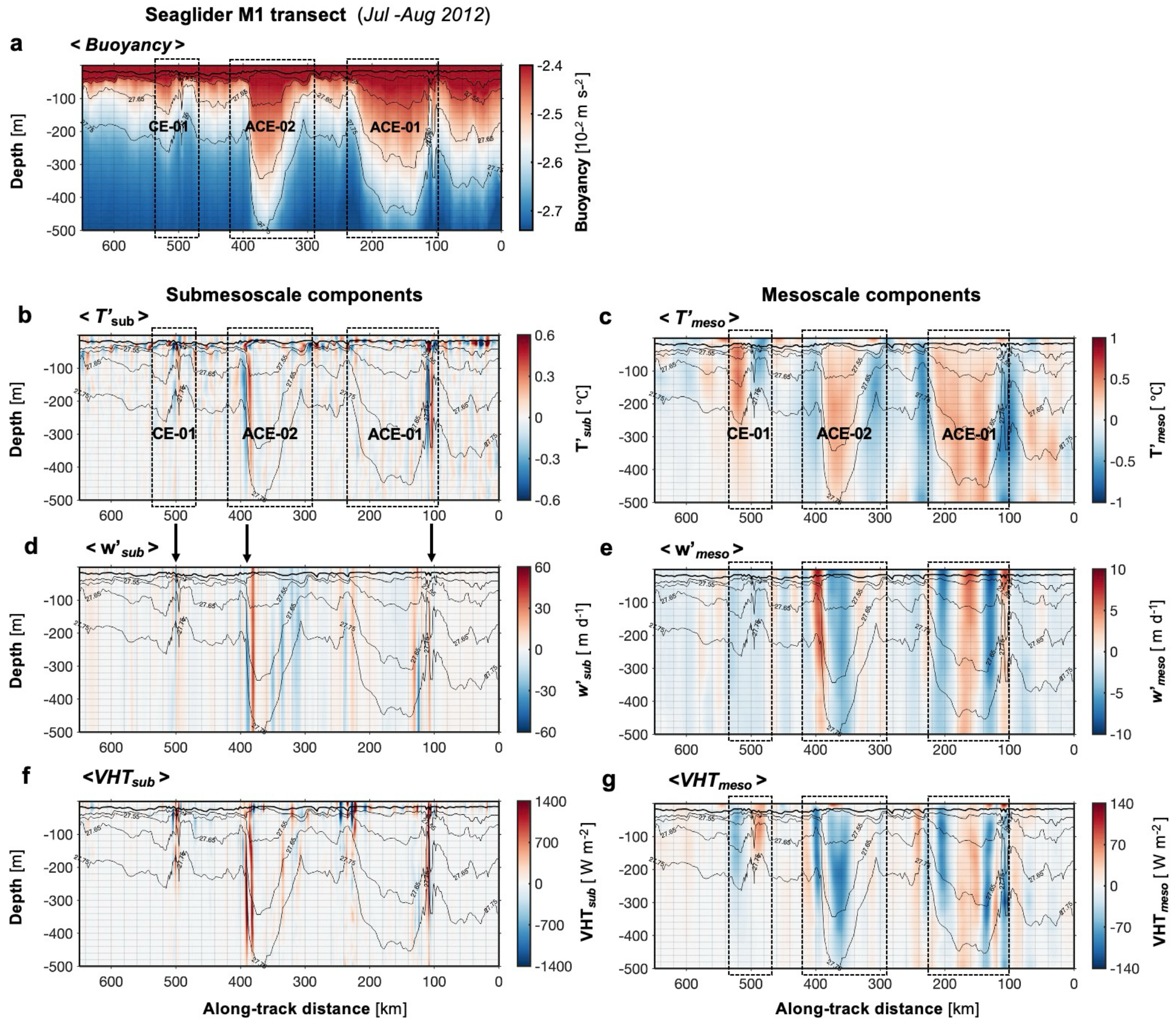

Supplementary Fig. S8 | Characteristics of submesoscale and mesoscale components along the Seaglider trajectory from 6 July 2012 to 16 August 2012. a The seaglider's vertical buoyancy section. **b** and **c** show temperature anomalies for the submesoscale and mesoscale, respectively; **d** and **e** show the corresponding anomalies in vertical velocity; and **f** and **g** illustrate vertical heat transport (VHT) for the submesoscale and mesoscale processes. The mixed layer depth (MLD) is marked by a thick black line in all panels.

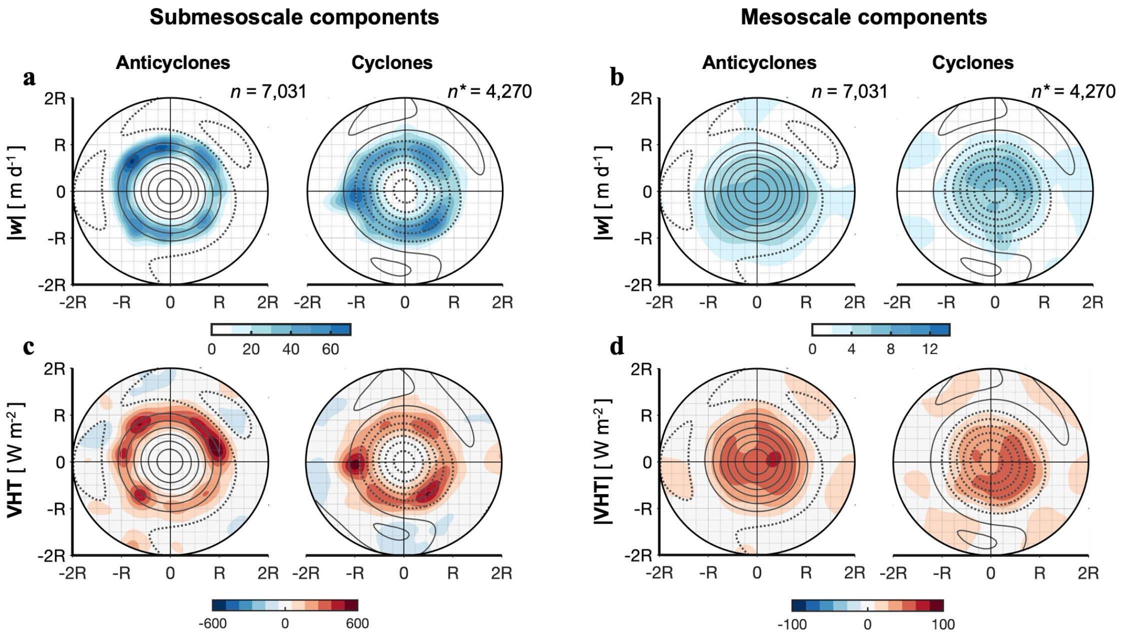

Supplementary Fig. S9 | Eddy-centric composite distributions in the normalized coordinate for submesoscale and mesoscale components (upper 500 m averages): a, b vertical velocity (|*w*|) and **c**, **d** vertical heat transport (VHT and |VHT|, respectively). Positive (negative) sea level anomalies (SLA) are represented by solid (dashed) black contours. *n* and *n** represent the number of Seaglider sampling profiles associated with anticyclonic and cyclonic eddies, respectively. *R* represents the normalized radius of the eddy.

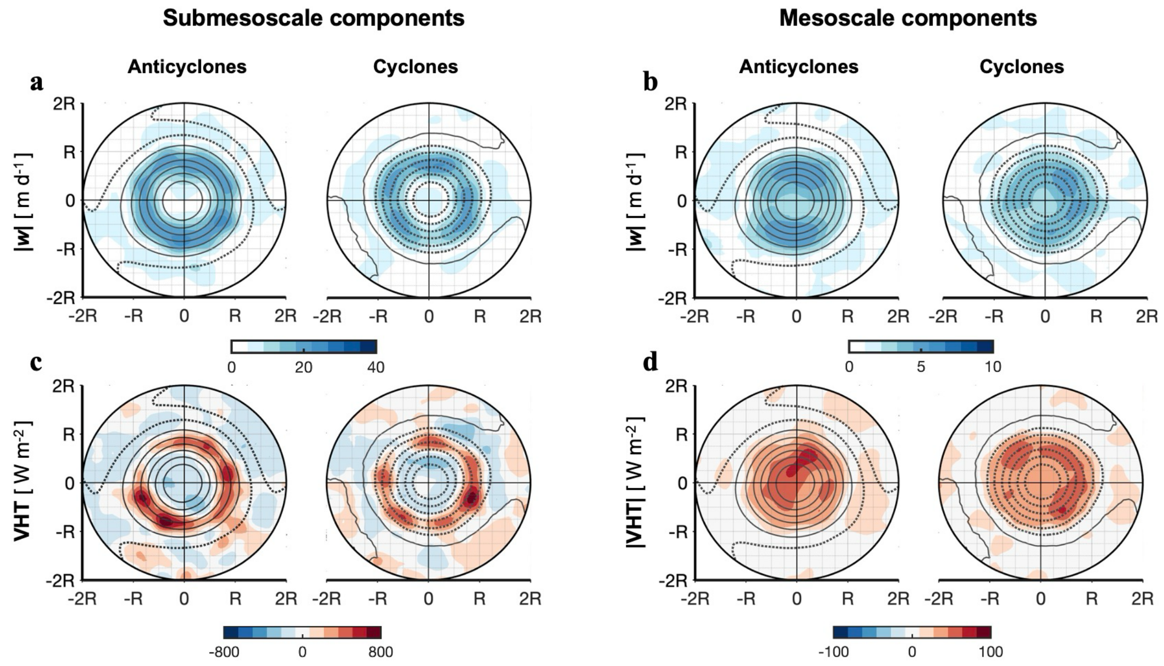

Supplementary Fig. S10 | Eddy-centric composite distributions of submesoscale and mesoscale components (averaged over the upper 500 m) in normalized coordinates from high-resolution numerical simulations during 1 December 2017 to 30 November 2019: a, b vertical velocity (|*w*|) and **c**, **d** vertical heat transport (VHT and |VHT|, respectively). Positive (negative) sea level anomalies (SLA) are represented by solid (dashed) black contours. *R* represents the normalized radius of the eddy.

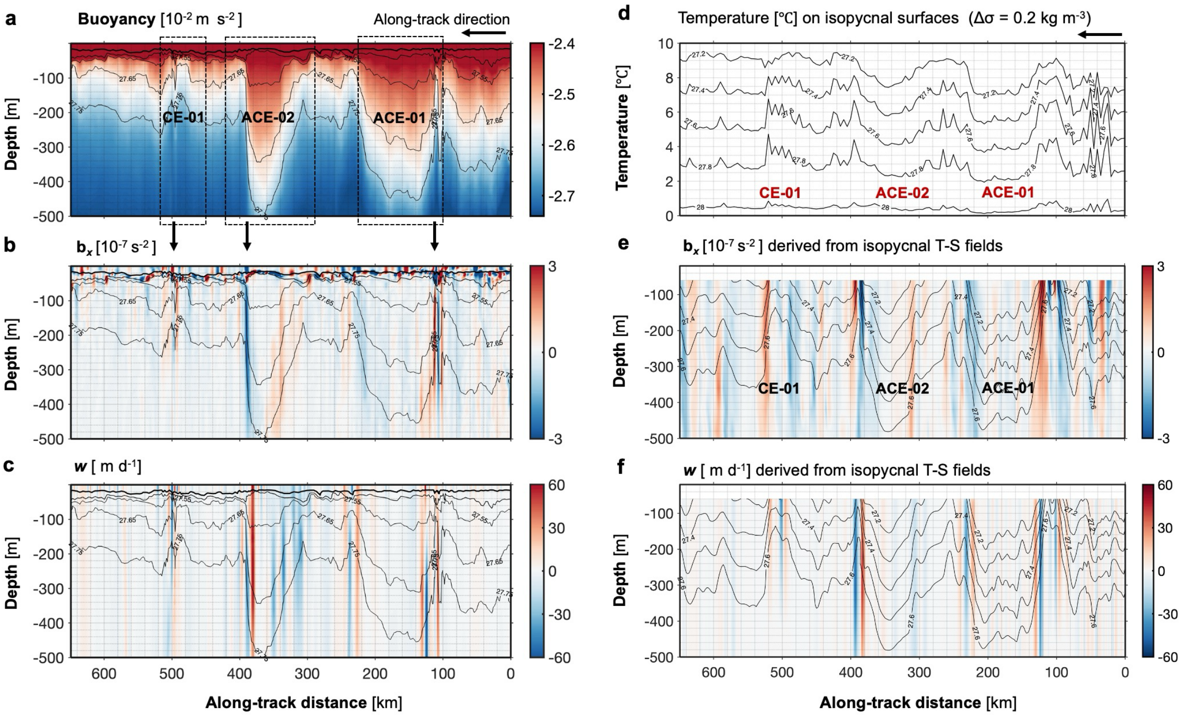

Supplementary Fig. 11 | a Buoyancy, **b** lateral buoyancy gradient (b_x), and **c** vertical velocity (w) in depth-distance coordinates (same as main text Figs. 2c, 2e, and 2g). **d** Potential temperature on selected isopycnal surfaces from the Seaglider transect, with isopycnal intervals of 0.2 kg m^{-3}; x-axis shows Seaglider along-track distance. **e** Lateral buoyancy gradient (b_x) and **f** vertical velocity (w) calculated in depth coordinates using isopycnal-filtered temperature and salinity data.

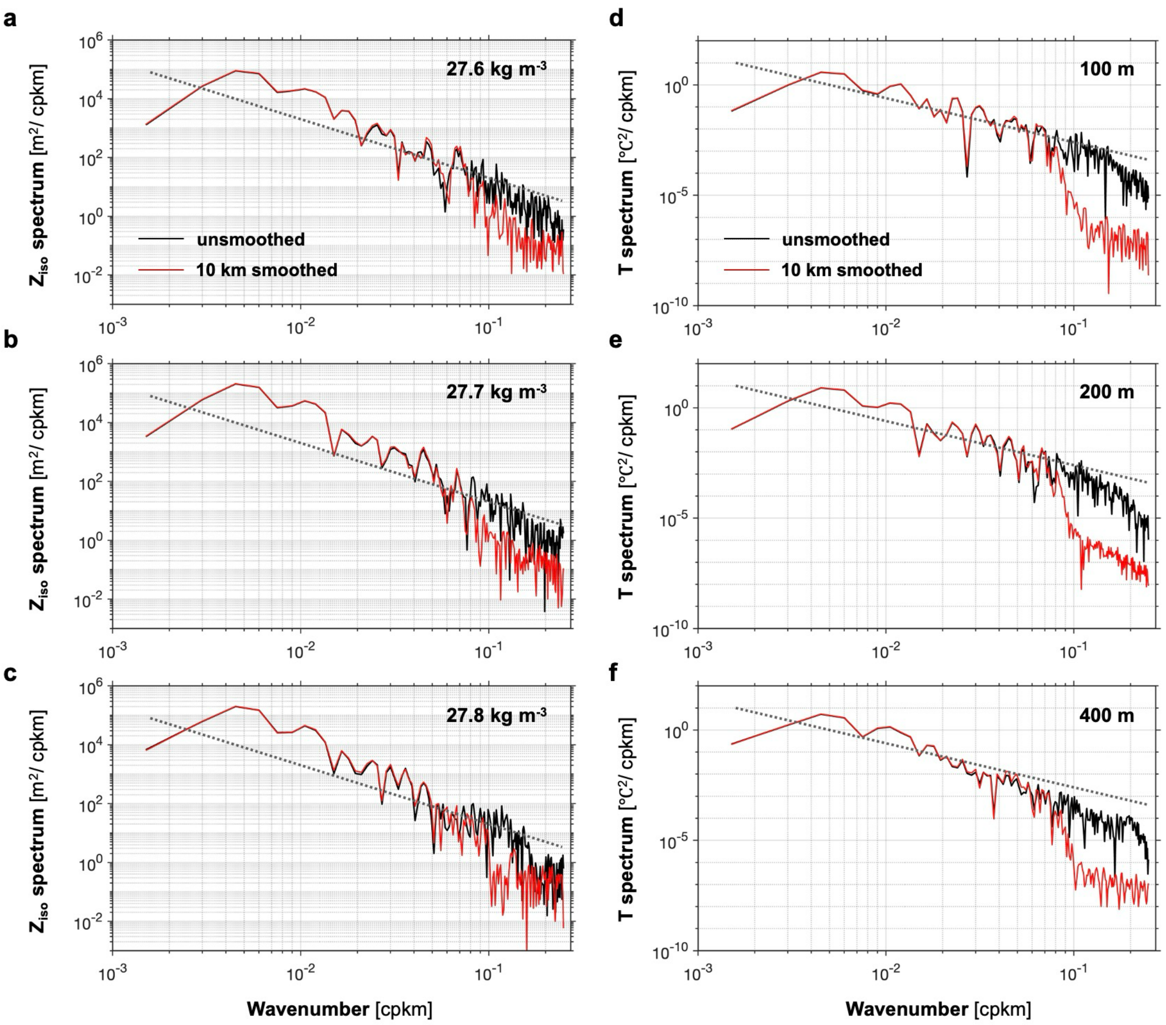

Supplementary Fig. 12 | Wavenumber spectra from the Seaglider transect (6 July 2012 to 16 August 2012) shown in main text Fig. 2. a-c Depth spectra for the 27.6, 27.7, and 27.8 kg m^{-3} isopycnal surfaces. **d-f** Potential temperature spectra at depths of 100 m, 200 m, and 400 m. In all panels, the black and red lines depict the spectra before and after applying a 10-km Gaussian filter, respectively. The dashed gray line indicates a k^{-2} slope for reference. Wavenumber is in cycles per kilometer (cpkm).

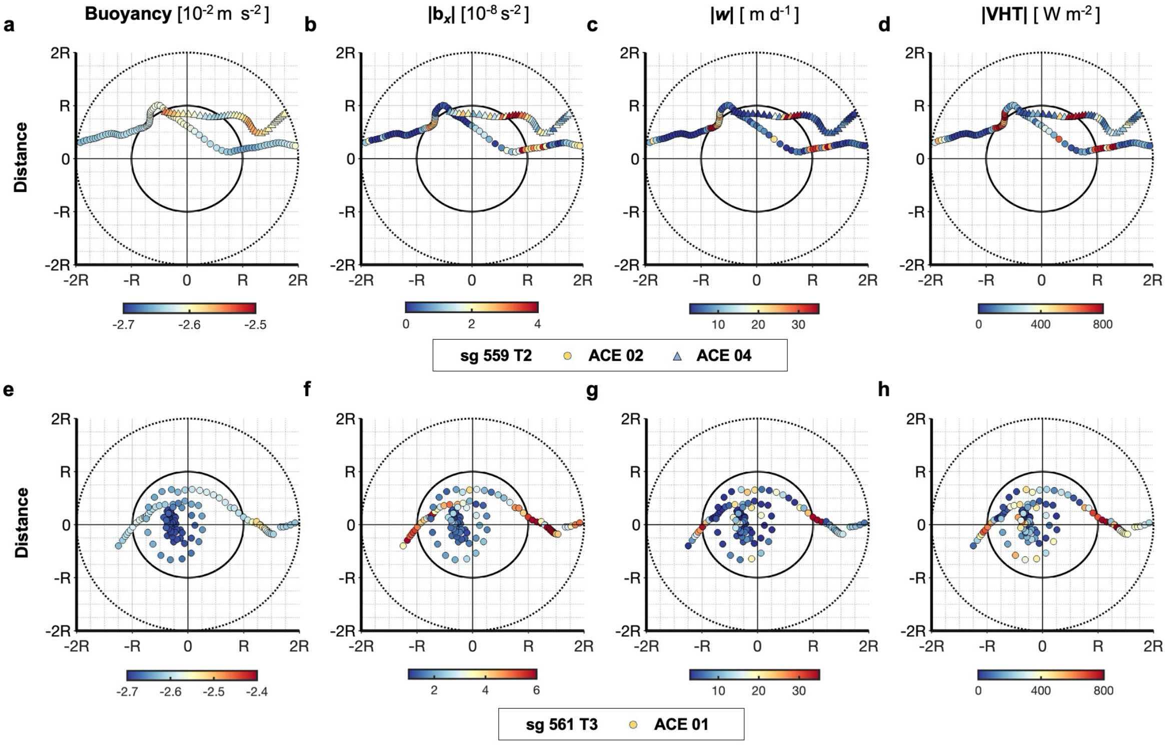

Supplementary Fig. 13 | Case study of Seaglider observations in normalized eddy-centric coordinates. a-d Seaglider sg559 transect T2 through ACE-02 and ACE-04 (see black dashed boxes in Fig. S3b): **a** buoyancy, **b** lateral buoyancy gradient (b_x), **c** vertical velocity (w), and **d** vertical heat transport (VHT). The circles and triangles denote the tracks of ACE-02 and ACE-04, respectively. **e-h** Seaglider sg561 transect T3 through ACE-01 (see black dashed box in Fig. S4b): **e** buoyancy, **f** lateral buoyancy gradient (b_x), **g** vertical velocity (w), and **h** VHT. All presented variables are averaged over the upper 500 m. R represents the normalized radius of the eddy.

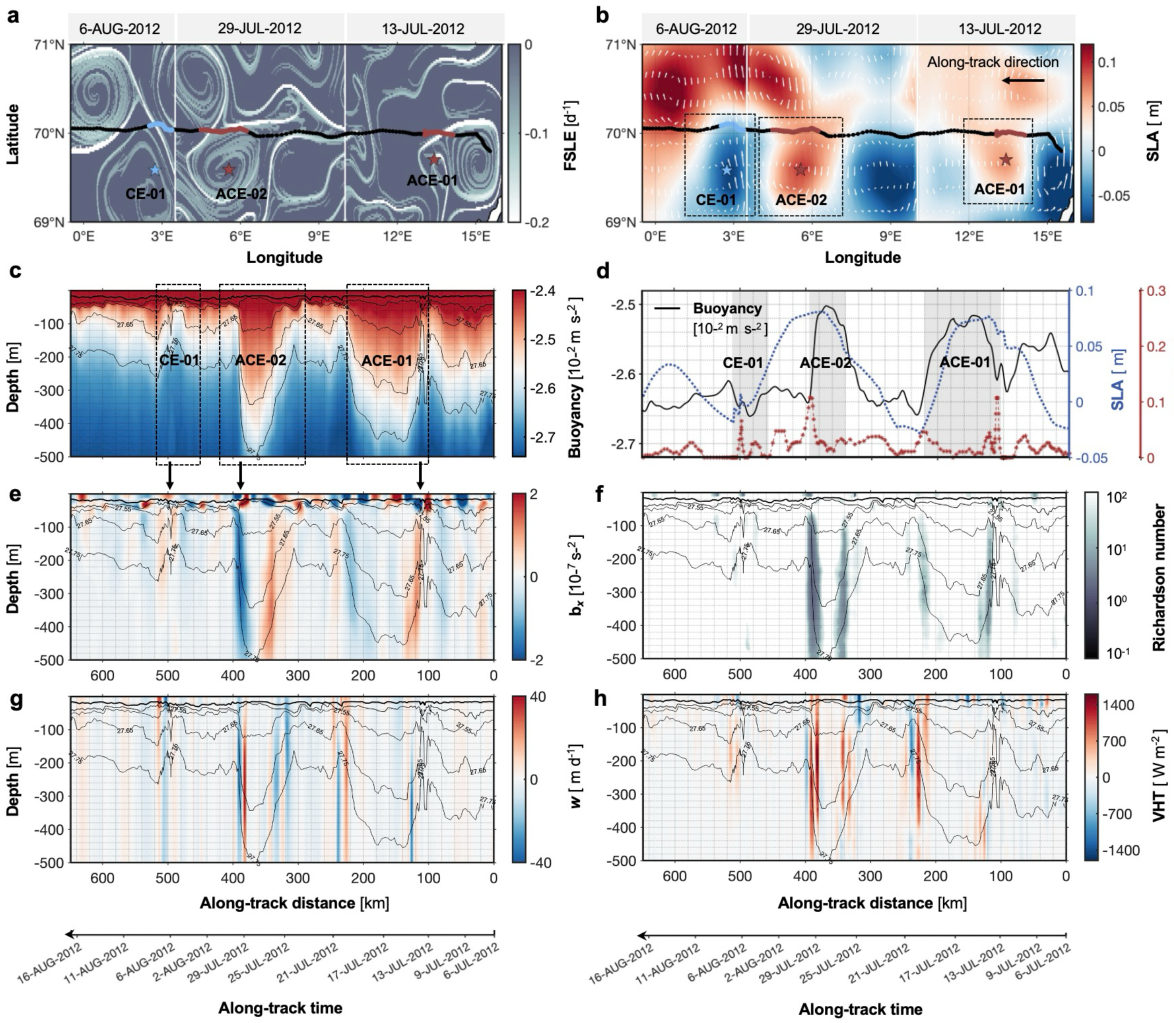

Supplementary Fig. 14 | The vertical section of all parameters is identical to those shown in Figure 2 of the main text, except here an objective mapping approach was applied in the analysis. **a** Finite-size Lyapunov exponent (FSLE) and **b** sea level anomaly (SLA) superimposed on the Seaglider track from 6 July 2012 to 16 August 2012. Seaglider vertical sections of **c** buoyancy, **e** lateral buoyancy gradient (b_x), **f** Richardson number (*Ri*), **g** vertical velocity (*w*), and **h** vertical heat transport (VHT), smoothed using a Gaussian window with a scale of 10 km. The mixed layer depth (MLD) is indicated by the thick black line in panels **c** and **e**–**h**. **d** Time series along the glider track showing averaged buoyancy (black line), SLA (blue line), and |FSLE| (red line).

Supplementary References

1. Hoskins, B. J. & Bretherton, F. P. Atmospheric frontogenesis models: Mathematical formulation and solution. *J. Atmospheric Sci.* **29**, 11–27 (1972).
2. Hoskins, B. J. The mathematical theory of frontogenesis. *Annu. review fluid mechanics* **14**, 131–151 (1982).
3. Hoskins, B. J., & Draghici, I. The forcing of ageostrophic motion according to the semi-geostrophic equations and in an isentropic coordinate model., *J. Atmos. Sci.* **34**, 1859–1867 (1977).
4. Hoskins, B. J., Draghici, I. & Davies, H. C. A new look at the ω-equation. *Q. J. R. Meteorol. Soc.* **104**, 31–38 (1978).
5. Hakim, G. & Keyser, D. Canonical frontal circulation patterns in terms of green's functions for the sawyer-eliassen equation. *Q. J. Royal Meteorol. Soc.* **127**, 1795–1814 (2001).
6. Garau, B., Ruiz, S., Zhang, W. G., Pascual, A., Heslop, E., Kerfoot, J., & Tintore, J. Thermal lag correction on Slocum CTD glider data. *J. Atmos. Oceanic Technol.* **28**, 1065-1071 (2011).
7. Frajka-Williams, E., Eriksen, C. C., Rhines, P. B., & Harcourt, R. R. Determining vertical water velocities from Seaglider. *J. Atmos. Oceanic Technol.* **28**, 1641-1656 (2011).
8. Eriksen, C. C., Osse, T. J., Light, R. D., Wen, T., Lehman, T. W., Sabin, P. L., Ballard, J. W., & Chiodi, A. M. Seaglider: A long-range autonomous underwater vehicle for oceanographic research. *IEEE J. Oceanic Eng.* **26**, 424–436 (2001).
9. Rudnick, D. L. Ocean research enabled by underwater gliders. *Annu. Rev. Mar. Sci.* **8**, 519–541 (2016).
10. Rudnick, D. L., & Cole, S. T. On sampling the ocean using underwater gliders. *J. Geophys. Res.* **116**, C08010 (2011).
11. Todd, R. E., Owens, W. B., & Rudnick, D. L. Potential vorticity structure in the North Atlantic western boundary current from underwater glider observations. *J. Phys. Oceanogr.* **46**, 327–348 (2016).
12. Bosse, A., & Fer, I. Mean structure and seasonality of the Norwegian Atlantic Front Current along the Mohn Ridge from repeated glider transects. *Geophys. Res. Lett.* **46**, 13,170–13,179 (2019).

13. Bosse, A., Testor, P., Damien, P., Estournel, C., Marsaleix, P., Mortier, L., et al. Wind-Forced Submesoscale Symmetric Instability around Deep Convection in the Northwestern Mediterranean Sea. *Fluids* **6**, 123 (2021).
14. Siegelman, L. et al. Enhanced upward heat transport at deep submesoscale ocean fronts. *Nat. Geosci.* **13**, 50–55 (2020).
15. Lévy, M., Franks, P. J. S. & Smith, K. S. The role of submesoscale currents in structuring marine ecosystems. *Nat. Commun.* **9**, 4758 (2018).
16. Su, Z., Wang, J., Klein, P., Thompson, A. F., & Menemenlis, D. Ocean submesoscales as a key component of the global heat budget. *Nat. Commun.* **9**, 775 (2018).
17. Cao, H., Jing, Z., & Fox ‑ Kemper, B. Scale-dependent vertical heat transport inferred from quasi-synoptic submesoscale-resolving observations. *Geophys. Res. Lett.* **51**, e2024GL110190 (2024).
18. Sperrevik, A. K., Röhrs, J., & Christensen, K. H. Impact of data assimilation on Eulerian versus Lagrangian estimates of upper ocean transport. *J. Geophys. Res. Oceans* **122**, 5445–5457 (2017).
19. Sperrevik, A.K., Christensen, K.H., & Röhrs, J. Constraining energetic slope currents through assimilation of high-frequency radar observations. *Ocean Sci.* **11**, 237–249 (2015).
20. Fairall, C. W., Bradley, E. F., Hare, J. E., Grachev, A. A., & Edson, J. B. Bulk parameterization of air-sea fluxes: updates and verification for the COARE algorithm. *J. Clim.* **16**, 571–591 (2003).
21. Xie, J., Bertino, L., Counillon, F., Lisæter, K. A., & Sakov, P. Quality assessment of the TOPAZ4 reanalysis in the Arctic over the period 1991–2013. *Ocean Sci.* **13**, 123–144 (2017).
22. Egbert, G. & Erofeeva, S. Efficient Inverse Modeling of Barotropic Ocean Tides. *J. Atmos. Ocean. Technol.* **19**, 183–204 (2002).
